# The association between Mental Health and Workaholism in Healthcare Workers

**DOI:** 10.1192/j.eurpsy.2025.1572

**Published:** 2025-08-26

**Authors:** K. Imen, R. Nakhli, L. S. Chabbi, O. Ajmi, M. Bouhoula, N. Belhadj, A. Aloui, M. Maoua, H. Kalboussi, O. El Maalel, S. Chatti, A. Chouchane, N. Mrizak

**Affiliations:** 1 Occupational Medicine Department, University of Sousse, Faculty of Medicine Ibn Al Jazzar, Sousse; 2 D Psychiatric Department, Razi Hospital, Tunis; 3 Psychiatry Department, Psychiatry Department, Farhat Hached University Hospital Medicine Department, Sahloul University Hospital; 4 Occupational Medicine Department, Occupational Medicine Department, Sahloul University Hospital, Sousse, Tunisia

## Abstract

**Introduction:**

Workaholism, or work addiction, is a compulsive drive to work excessively, often at the detriment of personal and professional well-being. This behavior is associated with increased risk of burnout and psychosocial disorders. While understudied in healthcare workers, the constant connectivity and demands of the healthcare profession can exacerbate workaholism. Occupational health teams must prioritize addressing this issue to mitigate its negative consequences.

**Objectives:**

To study the factors associated with workaholism among healthcare workers and to assess its impact on mental and physical well-being.

**Methods:**

A cross-sectional descriptive study examined the determinants of workaholism and its impact on quality of life among healthcare workers at the Farhat Hached University Hospital in Sousse, from March 2022 to June 2022. The Work Addiction Risk Test (WART) was used to assess workaholism and The Hospital Anxiety and Depression Scale (HAD) to measure mental health symptoms.

**Results:**

A total of 117 questionnaires were completed by healthcare workers, representing a response rate of 68.8%.The average age of participants was 35.45 years, with a female-dominated population (81.2%). Nurses comprised the largest group (73.5%), and most participants had less than 10 years of experience in their current department. Shift work was common, with 74.4% of participants working shifts. The Work Addiction Risk Test (WART) revealed that 18.2% of healthcare workers were at risk of work addiction, including 13.7% at moderate risk and 5.1% at high risk. The Hospital Anxiety and Depression Scale (HAD) indicated that 54.7% of participants suffered from anxiety and 35% from depression. A significant association was found between anxiety scores and a high risk of work addiction (p=0.003).

**Conclusions:**

This study highlights the potential for workaholism among healthcare workers, particularly those facing challenging work conditions such as shift work, heavy patient loads, staff shortages, and high levels of stress. Further studies with larger and more diverse samples are necessary to better understand the prevalence and impact of workaholism among healthcare professionals mental heath.

**Disclosure of Interest:**

None Declared

